# Protein kinase C regulates tonic GABA_A_ receptor-mediated inhibition in the hippocampus and thalamus

**DOI:** 10.1111/ejn.12352

**Published:** 2014-08-29

**Authors:** Damian P Bright, Trevor G Smart

**Affiliations:** Department of Neuroscience, Physiology & Pharmacology, University College LondonGower Street, London, WC1E 6BT, UK

**Keywords:** GABA_A_ receptor, mouse, PKC, tonic inhibition

## Abstract

Tonic inhibition mediated by extrasynaptic GABA_A_ receptors (GABA_A_Rs) is an important regulator of neuronal excitability. Phosphorylation by protein kinase C (PKC) provides a key mode of regulation for synaptic GABA_A_Rs underlying phasic inhibition; however, less attention has been focused on the plasticity of tonic inhibition and whether this can also be modulated by receptor phosphorylation. To address this issue, we used whole-cell patch clamp recording in acute murine brain slices at both room and physiological temperatures to examine the effects of PKC-mediated phosphorylation on tonic inhibition. Recordings from dentate gyrus granule cells in the hippocampus and dorsal lateral geniculate relay neurons in the thalamus demonstrated that PKC activation caused downregulation of tonic GABA_A_R-mediated inhibition. Conversely, inhibition of PKC resulted in an increase in tonic GABA_A_R activity. These findings were corroborated by experiments on human embryonic kidney 293 cells expressing recombinant α4β2δ GABA_A_Rs, which represent a key extrasynaptic GABA_A_R isoform in the hippocampus and thalamus. Using bath application of low GABA concentrations to mimic activation by ambient neurotransmitter, we demonstrated a similar inhibition of receptor function following PKC activation at physiological temperature. Live cell imaging revealed that this was correlated with a loss of cell surface GABA_A_Rs. The inhibitory effects of PKC activation on α4β2δ GABA_A_R activity appeared to be mediated by direct phosphorylation at a previously identified site on the β2 subunit, serine 410. These results indicate that PKC-mediated phosphorylation can be an important physiological regulator of tonic GABA_A_R-mediated inhibition.

## Introduction

GABA_A_ receptors (GABA_A_Rs) mediate two forms of inhibition within the brain, phasic and tonic (Farrant & Nusser, 2005). Phasic inhibition is mostly mediated by γ2 subunit-containing GABA_A_Rs located at inhibitory synapses that are activated by exocytotic GABA release, resulting in brief inhibitory postsynaptic currents (IPSCs). By contrast, tonic inhibition requires extrasynaptic α4/6δ or α5 subunit-containing GABA_A_Rs that are activated by ambient GABA concentrations, providing a persistent tonic membrane conductance. Tonic conductances are found in various brain regions, and are important for the control of neuronal excitability both *in vitro* (Brickley *et al*., 1996; Mitchell & Silver, 2003; Bright *et al*., 2007; Pavlov *et al*., 2009) and *in vivo* (Chadderton *et al*., 2004; Duguid *et al*., 2012). Indeed, dysfunctional tonic GABA_A_R-mediated inhibition is associated with various pathological conditions (Brickley & Mody, 2012), including, epilepsy (Zhan & Nadler, 2009), schizophrenia (Damgaard *et al*., 2011), depression (Merali *et al*., 2004), and fragile X syndrome (Olmos-Serrano *et al*., 2010).

Whereas various mechanisms that regulate the efficacy of synaptic inhibition have been identified (Olmos-Serrano *et al*., 2010; Cherubini, 2012), our knowledge regarding the modulation of tonic inhibition is far less complete. A key mode of regulation for synaptic GABA_A_Rs is via phosphorylation of substrate sites on β1–3 and γ2 subunits by both serine/threonine and tyrosine kinases (Moss *et al*., 1995; Vithlani *et al*., 2011). This covalent modification can directly change the functional properties of GABA_A_Rs, such as open probability (Moss *et al*., 1995) and desensitization kinetics (Jones & Westbrook, 1997), as well as altering their trafficking at inhibitory synapses (Vithlani *et al*., 2011). Indeed, kinases and phosphatases represent critical downstream effectors for various pathways that converge to modify inhibitory synaptic transmission (Luscher *et al*., 2011).

Recent studies have shown that specific extrasynaptic GABA_A_Rs responsible for tonic inhibition are also substrates for phosphorylation by serine/threonine kinases. Protein kinase C (PKC) phosphorylates a novel site, Ser443, on α4 subunits to enhance the surface stability of α4β3 GABA_A_Rs expressed in human embryonic kidney 293 (HEK293) cells and prevent GABA-activated current run-down (Abramian *et al*., 2010). In addition, Ca^2+^/calmodulin-dependent protein kinase II has been implicated in the regulation of extrasynaptic GABA_A_Rs (Houston *et al*., 2007; Saliba *et al*., 2012). Activation of L-type voltage-gated Ca^2+^ channels in cultured hippocampal neurons enhances Ca^2+^/calmodulin-dependent protein kinase II-mediated phosphorylation at a previously identified site on the β3 subunit, Ser383 (Houston *et al*., 2007), resulting in increased surface expression of α5β3γ2 GABA_A_Rs and increased tonic current.

Here, we examined tonic inhibitory plasticity and its regulation by phosphorylation in dentate gyrus granule cells (DGGCs), which provide a gateway to the hippocampal neural network, and in thalamic relay neurons within the dorsal lateral geniculate nucleus (dLGN), which form a key component of the visual system. Both populations of neurons express tonic inhibitory currents that are largely mediated by α4βδ GABA_A_Rs (Pirker *et al*., 2000; Cope *et al*., 2005; Bright *et al*., 2007; Glykys & Mody, 2007; Herd *et al*., 2008).

## Materials and methods

### Heterologous expression

HEK293 cells were cultured according to standard protocols, and were transfected 6 h after plating. A modified calcium phosphate method was used to introduce cDNAs encoding murine GABA_A_R α4, β2 and δ subunits. We used a super-ecliptic phluorin (SEP)-tagged δ subunit, which was created as previously described (Bright *et al*., 2011). Expression of the δ subunit was then assessed by surface fluorescence, altered Zn^2+^ sensitivity and GABA EC_50_ values relative to αβ expression alone (Bright *et al*., 2011). HEK293 cells were used for recording after a further 16–48 h.

### Acute slice preparations

Experiments were performed in compliance with the guidelines for the welfare of experimental animals issued by the European Communities Council Directive of 24 November 1986 (86/609/EEC). Brain slices were obtained from mature (> 1 month postnatal) male C57BL/6J mice, in accordance with the UK Animals (Scientific Procedures) Act 1986. All procedures have passed review by the UCL Ethical Review Committee. After terminal isoflurane anaesthesia, the brain was rapidly removed and then immersed in ice-cold slicing solution composed of 85 mm NaCl, 2.5 mm KCl, 1 mm CaCl_2_, 4 mm MgCl_2_, 1.25 mm NaH_2_PO_4_, 26 mm NaHCO_3_, 75 mm sucrose, and 25 mm glucose (pH 7.4 when bubbled with 95% O_2_ and 5% CO_2_). Horizontal slices containing the ventral hippocampus or coronal slices containing the dLGN (both 250 μm in thickness) were cut with a Leica VT1200S vibroslicer. Slices were incubated at 37 °C for 60 min, over which time the high-sucrose slicing solution was gradually replaced with normal recording solution containing 125 mm NaCl, 2.5 mm KCl, 2 mm CaCl_2_, 1 mm MgCl_2_, 1.25 mm NaH_2_PO_4_, 26 mm NaHCO_3_, and 25 mm glucose (pH 7.4 when bubbled with 95% O_2_ and 5% CO_2_).

### Electrophysiology – recombinant receptors

Cells were perfused with a recording solution, containing 140 mm NaCl, 2.5 mm CaCl_2_, 1.2 mm MgCl_2_, 4.7 mm KCl, 5 mm HEPES, and 11 mm glucose; the pH was adjusted to 7.4 with 1 m NaOH. Recordings were made either at room temperature (21–23 °C) or near-physiological temperature (34–36 °C). The solution temperature was monitored with a miniature thermocouple in the recording chamber. The pipette solution contained 140 mm CsCl, 2 mm NaCl, 0.5 mm CaCl_2_, 2 mm MgCl_2_, 10 mm HEPES, 5 mm EGTA, 2 mm Na-ATP, 0.5 mm Na-GTP, and 2 mm QX-314; the pH was adjusted to 7.3 with CsOH. Pipettes for whole-cell patch recording were pulled from thin-walled borosilicate glass (outer diameter, 1.5 mm; inner diameter, 1.17 mm; GC-150TF-10; Harvard Apparatus, Kent, UK), and had a resistance of 3–5 MΩ. Currents were recorded from green fluorescent protein-fluorescent cells with a Multiclamp 700B amplifier (Molecular Devices, California, USA). To reproduce the physiological activation of δ subunit GABA_A_Rs by ambient GABA concentrations within the brain, we applied low concentrations of GABA by bath perfusion. GABA-activated currents were allowed to reach steady state before application of other drugs. The following drugs were bath-applied: GABA (Sigma, Dorset, UK), phorbol 12-myristate 13-acetate (PMA) (Calbiochem, Middlesex, UK), and bisindolylmaleimide I (BIS-I) (Calbiochem).

### Electrophysiology – acute slice preparations

Hippocampal DGGCs and thalamic relay neurons in the dLGN were visually identified with a Nikon Eclipse F600N microscope equipped with differential interference contrast infrared optics. Thalamic relay neurons could be identified by their soma size and input resistance; other small cells with high input resistance were identified as local interneurons (Bright *et al*., 2007; Bright & Brickley, 2008). Slices were perfused with recording solution at a flow rate of 3–4 mL/min, and this, combined with the small volume of the recording chamber (approximately 800 μL), allowed for relatively fast solution exchange around the slice. Whole-cell recordings were acquired under voltage clamp with a Multiclamp 700B amplifier (Molecular Devices). Recording pipettes were fabricated as described for HEK293 cell recordings, and filled with the same internal solution. In some recordings, Lucifer yellow (2 mg/mL) or biocytin (4 mg/mL) was included in the internal solution to allow for later confocal imaging. Slices were fixed after recording in 4% paraformaldehyde, and in the case of biocytin-filled cells, processed on the following day with streptavidin–Alexa Fluor 555 (Invitrogen, Paisley, UK). Fluorescent neurons could then be visualized with a confocal microscope (Zeiss LSM 510). During whole-cell recording, GABA_A_R-mediated responses were pharmacologically isolated by inclusion of the ionotropic glutamate receptor blocker kynurenic acid (2 mm; Sigma) in the recording solution. The following drugs were bath-applied: bicuculline (BIC) (Sigma), tetrahydro-deoxycorticosterone (THDOC) (Sigma), zolpidem (Sigma), PMA, and BIS-I.

### Data analysis

Data acquisition was performed with pclamp9 (Molecular Devices). Current records were filtered at 2 kHz and digitized at 20 kHz with a Digidata 1440A (Molecular Devices). For all recordings, the series resistance, input resistance and capacitance were calculated from current responses to 10-mV hyperpolarizing voltage steps. In all cases, cells were recorded under control conditions for 5–10 min to allow stabilization of holding current and series resistance before application of drugs. For experiments involving pharmacological manipulation of kinase activity, effects on phasic and tonic inhibition were measured 15–20 min after drug application. For experiments involving neurosteroid (THDOC) or benzodiazepine (zolpidem) application, the drug onsets were much faster, and measurements were therefore made 4–5 min after drug application.

Synaptic events were analysed with winedr/winwcp software (John Dempster, University of Strathclyde, Glasgow, UK). Event detection was performed with amplitude-threshold crossing. Events were aligned on their initial rising phases, and averaged synaptic waveforms were constructed from IPSCs that showed monotonic rises and an uncontaminated decay phase (50–100 events). Average baseline current levels were calculated during a 10-ms epoch immediately prior to each detected event, and the peak amplitude was determined relative to this value. The 10–90% rise time was calculated between the start and the peak location, with interpolation between samples. The decay constant of individual IPSCs was calculated as the charge transfer during the baseline-corrected IPSC divided by the IPSC peak amplitude. This allowed a fit-independent estimate of the decay that could be determined for individual or averaged spontaneous IPSCs (sIPSCs). Phasic current was defined as the amount of charge carried by IPSCs per second, and was calculated by multiplying the average charge transfer during an IPSC by the average frequency.

The tonic GABA_A_R-mediated conductance was analysed in two ways. The tonic GABA_A_R-mediated current was calculated from the difference between the baseline current amplitude recorded before and that recorded after GABA_A_R blockade with 20 μm BIC. We also assessed the activity of tonically active GABA_A_Rs by measuring baseline root mean square (RMS) noise. This analysis was performed with winedr software. Recordings were divided into epochs containing 2048 samples (length, 1024 ms), and epochs containing synaptic currents were rejected either manually or by utilizing a running-threshold comparison in Excel. In this method, a running average (usually the median) of the RMS noise was calculated at 5-s intervals, and epochs were rejected according to a threshold set from this running average. This method was found to provide an efficient and reliable way of removing synaptic contamination. Subsequent analysis involved calculation of the mean (synaptic current-free) RMS noise over minute-long intervals and normalization to the interval immediately prior to drug application for each recording.

### Live cell imaging of recombinant δ–SEP GABA_A_Rs

HEK293 cells were transfected with cDNAs encoding murine GABA_A_R α4, β2 and δ–SEP subunits, as described above, and were then used for imaging after a further 16–48 h. Transfected cells were perfused with standard recording solution (as for the electrophysiology experiments) at 30–32 °C, and imaged with a Zeiss Axioskop LSM510 confocal microscope equipped with an Achroplan ×40 water immersion differential interference contrast objective (numerical aperture, 0.8). Solutions containing GABA (300 nm) and GABA + PMA (200 nm) were bath applied during imaging. Fluorescence from the δ–SEP subunit was revealed following excitation with a 488-nm argon laser, and detected through a 505–530-nm bandpass filter. Images were acquired as the mean of four scans in eight bits, with the confocal settings (detector gain, amplifier offset, and laser intensity) optimized at *t* = 0 and then remaining unaltered for subsequent time points. During imaging experiments, transmitted light images were also captured to ensure that there were no significant changes in cell morphology. To assess surface fluorescence of the δ–SEP subunit, we applied our standard recording solution, adjusted to pH 4.5 with HCl, to quench the fluorescence of the SEP moiety (Ashby *et al*., 2004). Confocal images were analysed with imagej (version 1.45s) (National Institutes of Health, Bethesda, MD, USA). The mean fluorescence was determined for a region of interest (ROI) centred at the cell surface. Background fluorescence was set by imaging a region of the coverslip devoid of cells. This was subtracted from the fluorescence for the cell surface ROI, yielding a mean background-corrected fluorescence. For drug application experiments, fluorescence was normalized to the point at which PMA was applied (5 min after GABA application).

### Statistical analysis

Statistical tests were performed with prism (GraphPad Software, California, USA). Differences between groups were examined with the appropriate paired or unpaired Student∼s *t*-test, except where indicated, and were considered significant at *P* < 0.05. For comparisons of RMS noise during prolonged recordings, we used pooled raw data and compared them with the time point immediately prior to drug application, using paired *t*-tests.

## Results

### Tonic inhibition of DGGCs and dLGN relay neurons

**Fig. 1:**
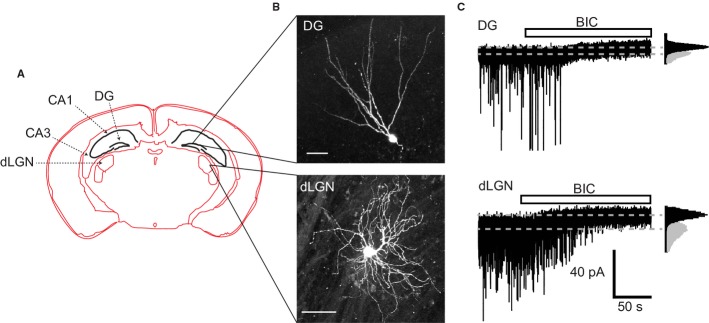
Properties of DGGCs and dLGN relay neurons. (A) Schematic diagram of a coronal mouse brain section, indicating the locations of hippocampal CA1, CA3 and DG regions, as well as the thalamic dLGN. (B) Confocal images (Z-projections of image stacks) of typical DGGCs (top panel) and dLGN relay neurons (bottom panel). Scale bars: 50 μm. (C) Representative membrane currents from DGGCs (top) and dLGN relay neurons (bottom). BIC (20 μm) reveals the presence of a tonic GABA_A_R-mediated conductance, as well as blocking sIPSCs. Insets: all-points histograms in controls (grey) and after BIC application (black).

Whole-cell recordings were made from visually identified hippocampal granule cells within the dentate gyrus (DG) and thalamic relay neurons within the dLGN. Both of these nuclei were easily identified within the slice preparation (Fig. 1A). Some cells were filled with either a fluorescent dye (Lucifer yellow) or biocytin (see Materials and methods) to allow for later confocal imaging to check neuronal location and morphological properties. Representative images of a DGGC and a dLGN relay neuron are shown in Fig. 1B. In each cell type, application of the GABA_A_R antagonist BIC (20 μm) abolished sIPSCs (Fig. 1C) and reduced the holding current and baseline noise, in accord with the presence of a tonic GABA_A_R-mediated conductance.

### Characterizing tonic and phasic inhibition in DGGCs at room and physiological temperature

**Fig. 2:**
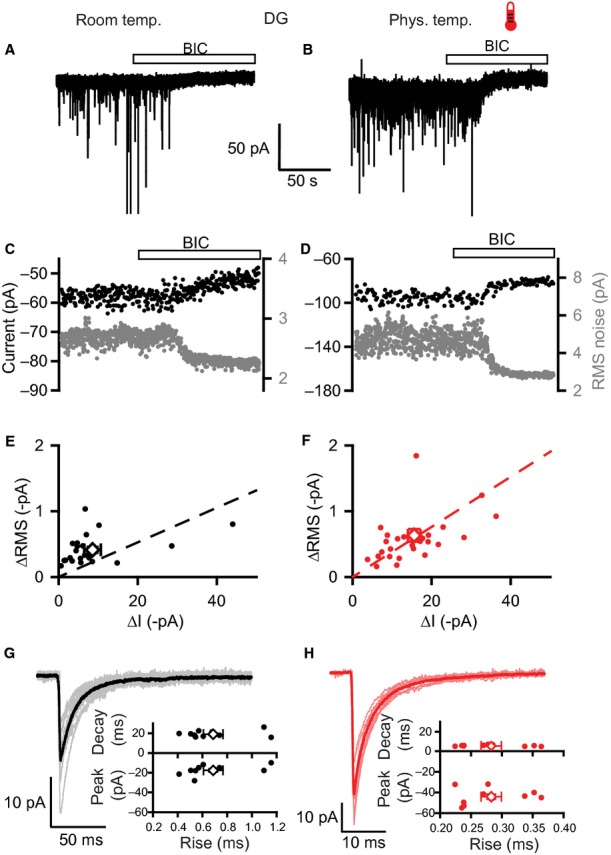
Properties of tonic and phasic inhibition in DGGCs at room and physiological temperature. (A and B) Membrane currents recorded from DGGCs at room (A) and physiological (B) temperatures (thermometer symbol). Bicuculline (BIC; bar) blocks the sIPSCs and a steady-state inward current with a reduction in the baseline noise, revealing the presence of a tonic GABA_A_ receptor-mediated conductance. (C and D) Plots of holding current (black circles) and RMS noise (grey) for the same experiments depicted in (A) and (B), over the same timescale. (E and F) Scatter plots of changes to holding currents (ΔI) and RMS noise (ΔRMS) by bicuculline for recordings at room (E) and physiological (F) temperatures. Open symbols and error bars are mean values ± SEMs. Linear regression analyses (dashed lines) illustrate positive correlations between changes in holding current and RMS noise. (G and H) Properties of sIPSCs at room (G) and physiological (H) temperatures. Average IPSC waveforms were constructed for 10 cells at each temperature (thin lines) along with superimposed global average IPSC waveforms (bold lines). Scatter plots show the mean values of peak amplitude, decay time and 10–90% rise time for these 10 cells with open symbols indicating global averages.

We recorded from mature (mean, 54 days; range, 30–100 days) DGGCs at both room temperature (21–23 °C, 343 cells) and near-physiological temperature (34.8 ± 0.1 °C, 191 cells). At each temperature, BIC (20 μm) reduced the holding current (Fig. 2A, B, E, and F: room temperature, 8.6 ± 2.1 pA, *n* = 22; physiological temperature, 15.6 ± 1.6 pA, *n* = 27). When normalized to cell capacitance, this corresponds to tonic GABA_A_R-mediated conductances of 2.8 ± 0.7 pS/pF (room temperature) and 4.3 ± 0.5 pS/pF (physiological temperature). Concurrent with the change in holding current, BIC also reduced the RMS baseline noise (Fig. 2C–F: room temperature, 0.41 ± 0.05 pA, *n* = 22; physiological temperature, 0.63 ±0.09 pA, *n* = 27). Notably, the change in holding current was closely correlated with the change in RMS noise at both temperatures (Fig. 2E and F, Pearson∼s correlation coefficients: room temperature, *R* = 0.54, *P* = 0.01; physiological temperature, *R* = 0.48, *P* = 0.01).

sIPSCs were confirmed as being mediated by GABA_A_Rs following blockade by BIC (Fig. 2A and B). Their properties were examined by constructing mean IPSC waveforms (see Materials and methods; Fig. 2G and H). At physiological temperature, sIPSCs were larger, showing faster rise and decay time kinetics, and occurred more frequently than at room temperature (Fig. 2G and H; Table 1).

**Table 1:** Properties of IPSCs in DGGCs at room and physiological temperatures

	Recording conditions	Peak amplitude (pA)	10–90% rise time (ms)	Decay time (ms)	Frequency (Hz)	*n*
sIPSCs	Room temperature (21–23 °C)	−17.6 ± 1.6	0.69 ± 0.08	19.0 ± 1.0	1.0 ± 0.2	10
	Physiological temperature (34–37 °C)	−44.2 ± 2.4	0.28 ± 0.02	5.8 ± 0.2	3.8 ± 0.8	10
mIPSCs	Room temperature (21–23 °C)	−15.6 ± 1.8	0.34 ± 0.04	11.2 ± 0.9	0.8 ± 0.2	8

### Using RMS noise to measure tonic GABA_A_R-mediated inhibition

Our recordings in DGGCs showed that the amplitude of the tonic GABA_A_R-mediated current was relatively small, particularly at room temperature (Fig. 2E and F). This is in accord with previous studies demonstrating small tonic currents, unless the amount of GABA in the slice is increased by either blocking GABA uptake or by adding GABA to the recording solution (Nusser & Mody, 2002; Naylor *et al*., 2005; Mtchedlishvili & Kapur, 2006; Zhan & Nadler, 2009). Such tonic currents will be subject to measurement error, but changes in RMS current noise may offer greater reliability for monitoring the activity of tonically active GABA_A_Rs (Mtchedlishvili & Kapur, 2006; Glykys & Mody, 2007). To test the feasibility of using RMS noise as a more reliable monitor for tonic GABA_A_R activity in DGGCs, we used low concentrations of two modulators: the neurosteroid THDOC, and the benzodiazepine zolpidem.

**Fig. 3:**
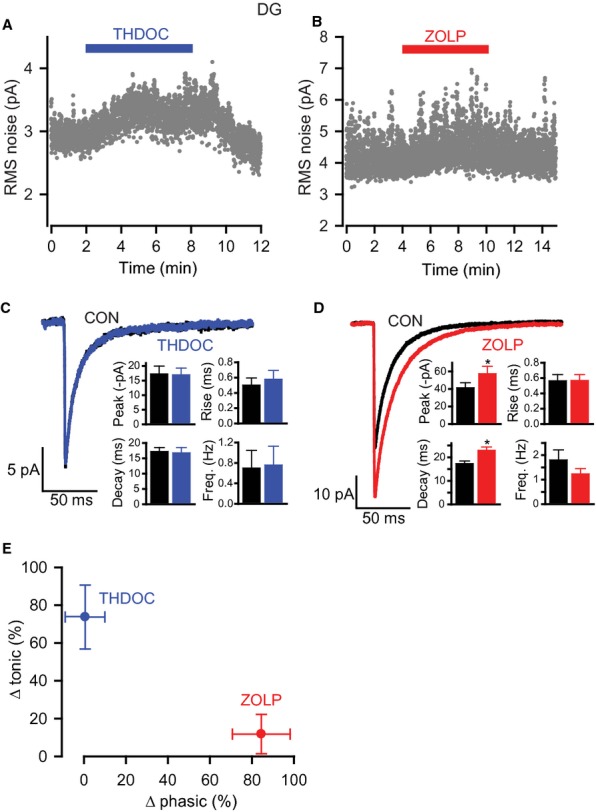
Effects of THDOC and zolpidem on RMS noise and sIPSCs in DGGCs. (A and B) RMS noise-time plots showing the effects of 50 nm THDOC and 50 nm zolpidem. (C and D) Analysis of sIPSCs in the absence and presence of THDOC (C) or zolpidem (D). Average IPSC waveforms from representative cells were constructed for controls and in the presence of drug. Bar charts compare mean sIPSC properties (peak amplitude, 10–90% rise time, decay time and frequency) for control, THDOC and zolpidem for all cells recorded (n = 6 for both experiments, *P < 0.05). (E) Summary plot showing changes in phasic (Δ phasic) and tonic (Δ tonic) inhibition caused by THDOC and zolpidem. The changes in phasic and tonic inhibition are quantified as the change in current carried by sIPSCs and the change in the GABA_A_ receptor-dependent component of the RMS noise, respectively. Symbols are means ± SEM. ZOLP, zolpidem.

Application of 50 nm THDOC caused a small, but significant, increase in RMS noise without a discernible effect on the holding current (Fig. 3A: change in RMS noise, 0.22 ± 0.03 pA, *n* = 9, *P* = 4.4 × 10^−5^; change in holding current, −2.5 ± 1.6 pA, *P* = 0.16). At this concentration, THDOC had no effect on GABA_A_Rs underlying synaptic inhibition, as shown by the lack of any change in sIPSC frequency, amplitude, or kinetics (Fig. 3C; Table 2). In contrast, zolpidem (50 nm) significantly increased sIPSC peak amplitude and prolonged the decay time (with no effects on rise time or frequency; Fig. 3D; Table 2). However, consistent with a lack of effect on extrasynaptic GABA_A_Rs underpinning the tonic conductance (Nusser & Mody, 2002; Mtchedlishvili & Kapur, 2006), zolpidem altered neither RMS noise (Fig. 3B: zolpidem, 3.20 ± 0.32 pA; control, 3.13 ± 0.28 pA; *n* = 6, *P* = 0.21) nor the holding current (zolpidem, −86.0 ± 5.3 pA; control, −88.6 ± 5.1 pA; *P* = 0.64).

**Table 2:** Effects of drugs on IPSC properties in DGGCs

	Recording temperature	Peak amplitude (pA)	10–90% rise time (ms)	Decay time (ms)	Frequency (Hz)	*n*
sIPSCs						
Control		−17.4 ± 2.6	0.50 ± 0.09	17.3 ± 1.2	0.7 ± 0.3	
THDOC 50 nm	Room	−17.1 ± 2.2	0.58 ± 0.11	16.9 ± 1.7	0.8 ± 0.4	6
Control		−41.6 ± 5.6	0.57 ± 0.08	17.4 ± 1.0	1.8 ± 0.4	
Zolpidem 50 nm	Room	−57.7 ± 8.2*	0.57 ± 0.07	23.1 ± 1.3*	1.2 ± 0.2	6
Control		−19.1 ± 2.5	0.73 ± 0.10	20.8 ± 1.5	1.0 ± 0.4	
PMA 200 nm	Room	−20.6 ± 3.4	0.63 ± 0.12	21.2 ± 0.9	0.8 ± 0.3	6
Control		−44.3 ± 3.6	0.27 ± 0.02	6.3 ± 0.2	2.6 ± 0.6	
PMA 200 nm	Physiological	−46.6 ± 7.2	0.35 ± 0.07	6.4 ± 0.3	2.9 ± 0.6	9
Control		−35.5 ± 4.0	0.67 ± 0.11	18.2 ± 1.1	1.4 ± 0.3	
BIS-I 500 nm	Room	−40.9 ± 4.8	0.60 ± 0.11	19.8 ± 0.6	0.9 ± 0.1	7
Control		−38.7 ± 4.0	0.36 ± 0.05	7.0 ± 0.6	7.0 ± 1.2	
BIS-I 500 nm	Physiological	−40.9 ± 4.8	0.30 ± 0.02	7.1 ± 0.4	6.8 ± 1.4	7
mIPSCs						
Control		−15.6 ± 1.8	0.34 ± 0.04	11.2 ± 0.9	0.8 ± 0.2	
PMA 200 nm	Room	−18.7 ± 1.9*	0.32 ± 0.03	12.0 ± 0.5	0.9 ± 0.1	8

Room temperature is 21–23 °C and physiological temperature is 34–37 °C. The only significant differences from control conditions are for the peak amplitude and decay time of sIPSCs in 50 nm zolpidem and for the peak amplitude of mIPSCs in 200 nm PMA.

* *P* < 0.05, paired *t*-test.

By quantifying the actions of these drugs on tonic inhibition, from calculating the effect on the GABA_A_R-mediated component of the RMS noise (i.e. by normalization to the BIC-sensitive component), we found that 50 nm THDOC caused a significant increase in tonic inhibition but had no effect on phasic inhibition (Fig. 3E: change in tonic inhibition, 73.8 ± 16.9%, *P* = 0.0012; change in phasic inhibition, 0.6 ± 9.3%, *P* = 0.45). In contrast, zolpidem significantly increased phasic inhibition (quantified as the phasic current; see Materials and methods), with no change in tonic inhibition (Fig. 3E: change in phasic inhibition, 84.5 ± 13.8%, *P* = 0.04; change in tonic inhibition, 11.8 ± 10.4%, *P* = 0.31). Therefore, baseline RMS noise is a sensitive and selective indicator of changes in the small tonic inhibitory conductances in these cells.

### Activation of PKC decreases tonic GABA_A_R activity in DGGCs

**Fig. 4:**
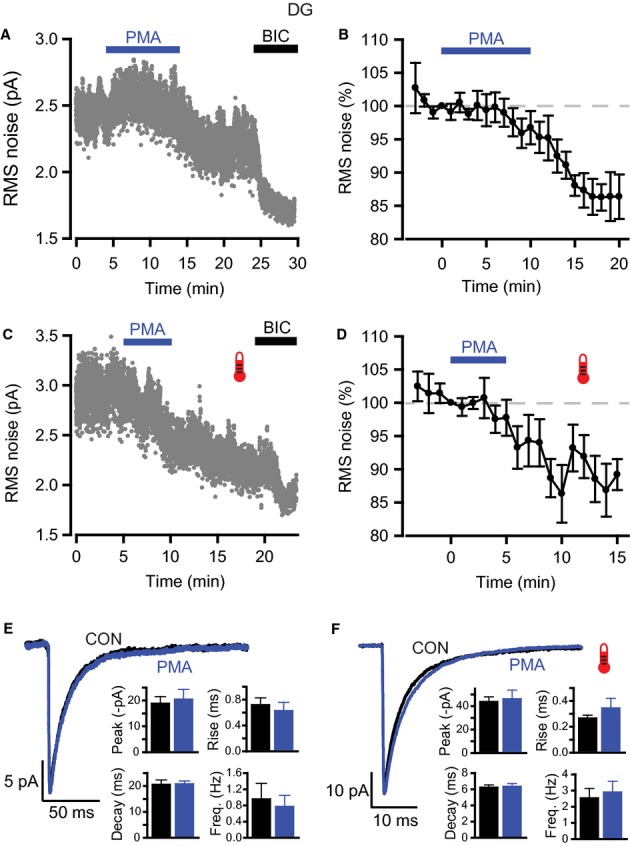
Effects of PKC activation on GABA_A_ receptor-mediated inhibition in DGGCs. (A and C) RMS noise-time plots for typical recordings during the application of PMA (200 nm) and bicuculline (20 μm) at room (A) and physiological (C) temperatures. PMA gradually reduced RMS noise, with bicuculline blocking a residual GABA_A_ receptor-dependent component of the noise. (B and D) Pooled data showing the effects of PMA on RMS noise at room (B, *n* = 6) and physiological (D, *n* = 9) temperature. RMS noise was averaged over 60 s epochs and then normalized to the epoch just prior to PMA application (see Materials and methods). (E and F) Average IPSC waveforms constructed for control and after PMA at room (E) and physiological (F) temperature. Bar charts show the lack of effect of PMA on sIPSC parameters (peak amplitude, 10–90% rise time, decay time and frequency).

Previous studies have shown that GABA_A_R subunits are substrates for phosphorylation by PKC, and that currents mediated by these receptors are differentially regulated by this kinase (Krishek *et al*., 1994; Lin *et al*., 1994; Poisbeau *et al*., 1999; Brandon *et al*., 2000; Abramian *et al*., 2010). We used the specific PKC activator PMA to investigate whether PKC regulates tonic GABA_A_ inhibition in DGGCs. PMA (200 nm) was bath applied to hippocampal slices at both room and physiological temperatures. To avoid prolonged application having deleterious effects on slice health, we applied PMA for either 10 min (room temperature) or 5 min (physiological temperature) before returning slices to control solution. With this protocol, PMA initiated a progressive reduction in baseline RMS noise at both temperatures (Fig. 4A–D). The onset was relatively fast, with the noise declining 5–10 min after PMA application at room temperature and after only a few minutes at physiological temperature. Although small, the reductions were significant (Table 3: room temperature, control, 2.60 ± 0.14 pA, at 20 min after PMA application, 2.26 ± 0.16 pA, equivalent to a 13.6 ± 3.3% reduction, *n* = 6, *P* = 0.014; physiological temperature, control, 3.42 ±0.37 pA, at 10 min after PMA application, 2.77 ± 0.28 pA, equivalent to a 13.7 ± 4.3% reduction, *n* = 9, *P* = 0.006). Application of BIC (20 μm) at the end of the recording caused a further decrease in RMS noise, indicating that there was still a component of the current noise mediated by GABA_A_R activity after the PMA-evoked reduction (Fig. 4A and C).

**Table 3:** RMS noise for DGGCs and dLGN relay neurons

Cell type	Treatment	Recording temperature	Control (pA)	Drug (pA)	BIC (pA)	*n*
DGGC	PMA	Room	2.60 ± 0.14	2.26 ± 0.16*	1.77 ± 0.16*	6
		Physiological	3.42 ± 0.37	2.77 ± 0.28*	2.08 ± 0.12*	9
	BIS-I	Room	3.22 ± 0.31	3.49 ± 0.35*	2.43 ± 0.28*	7
		Physiological	3.28 ± 0.23	3.83 ± 0.40*	2.87 ± 0.27*	7
	PMA in BIC	Room	2.45 ± 0.23	2.33 ± 0.9	–	5
	BIS-I in BIC	Room	2.25 ± 0.10	2.26 ± 0.11	–	9
dLGN	PMA	Room	5.76 ± 0.74	5.13 ± 0.60*	2.90 ± 0.23*	8
	BIS-I	Room	5.04 ± 0.59	5.73 ± 0.73*	3.38 ± 0.31*	8
	PMA in BIC	Room	3.36 ± 0.27	3.40 ± 0.37	–	6
	BIS-I in BIC	Room	3.17 ± 0.25	3.06 ± 0.24	–	5

Control measurements were made just prior to drug application, drug measurements were made 15–20 min after drug application, and BIC measurements were made 4–5 min after BIC application. For the BIC pre-application experiments, the control value was measured in BIC just prior to drug application.

* *P* < 0.05 (paired *t*-test), significant differences as compared with control values.

To ensure the accuracy of these measurements, we used several controls. First, to check that this reduction in RMS noise was mediated via an action on GABA_A_Rs, PMA was applied in the presence of BIC (20 μm) to block all GABA_A_R activity. Under these conditions, there was no reduction in RMS noise (Table 3: room temperature, 2.0 ± 3.4% reduction in RMS noise, *n* = 5, *P* = 0.59). Second, we checked the stability of RMS noise over a prolonged period in the absence of drugs. Indeed, there were no significant changes in RMS noise at room temperature (normalized noise, 99.8 ± 0.8%, *n* = 5, *P* = 0.29) or physiological temperature (normalized noise, 103.5 ± 4.4%, *n* = 5, *P* = 0.48) over a period of 25–30 min. Third, we checked whether changes in series resistance (*R*_s_) could have caused a spurious change in RMS noise during the recordings with PMA. Reassuringly, *R*_s_ remained stable during these recordings (room temperature, control, 14.7 ± 1.8 MΩ, after PMA application, 15.1 ± 1.5 MΩ; physiological temperature, control, 12.9 ± 0.9 MΩ, after PMA application, 13.5 ± 0.9 MΩ). Therefore, the reduction in baseline noise after PMA application represents a genuine decrease in tonic GABA_A_R activity.

We then constructed average sIPSC waveforms before and after PMA application to determine whether there was any effect of PKC activation on phasic inhibition under our conditions. However, the amplitude, kinetics and frequency of sIPSCs remained unchanged following PMA application at both recording temperatures (Fig. 4E and F; Table 2). However, PKC can affect miniature IPSC (mIPSC) amplitudes in DGGCs (Poisbeau *et al*., 1999). Therefore, recordings were also made in the presence of 500 nm tetrodotoxin to evaluate the impact of PKC on mIPSCs. Average mIPSC waveforms constructed under control conditions at room temperature revealed that mIPSCs showed a similar peak amplitude and frequency to sIPSCs, but with faster kinetics (Table 1). After application of PMA (200 nm), mIPSC frequency and kinetics remained unchanged (Table 2), but, consistent with the earlier study, mIPSC peak amplitude was increased (PMA, −18.7 ± 1.9 pA; control, −15.6 ± 1.8 pA; *n* = 8, *P* = 0.04).

### Inhibition of PKC increases tonic GABA_A_R activity in DGGCs

**Fig. 5:**
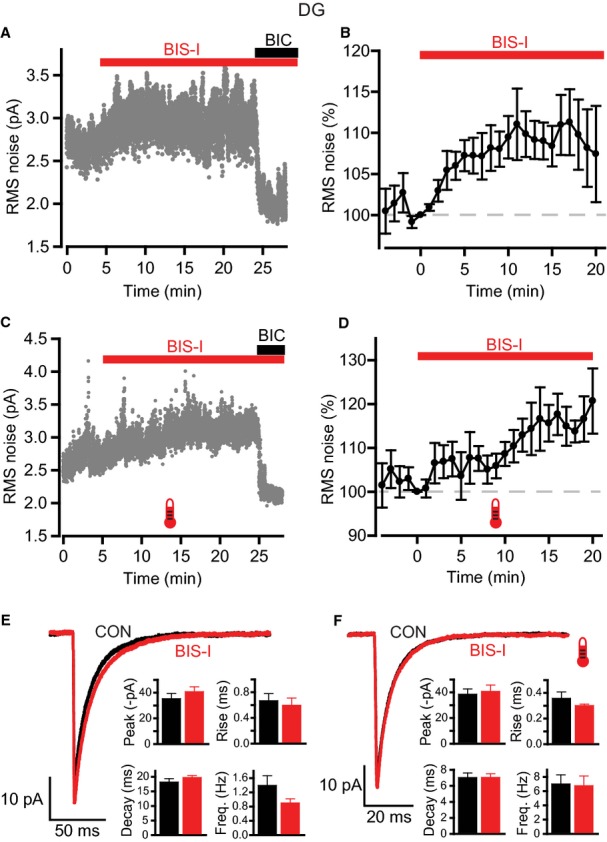
Effects of PKC inhibition on GABA_A_ receptor-mediated inhibition in DGGCs. (A and C) RMS noise-time plots showing the effects of bisindolylmaleimide I (BIS-I; 500 nm) and bicuculline (20 μm) during typical recordings at room (A) and physiological (C) temperatures. BIS-I increases RMS noise, whilst co-application of bicuculline at the end of the recording blocks this elevation and causes a reduction to below control levels. (B and D) Time courses for normalized data pooled from all recordings at room (B, *n* = 7) and physiological (D, *n* = 7) temperatures. (E and F) Application of BIS-I had little effect on sIPSCs. Average IPSC waveforms were constructed in control and after BIS-I; representative examples are shown for recordings at room (E) and physiological (F) temperature. Insets: bar charts for sIPSC parameters (peak amplitude, 10–90% rise time, decay time and frequency).

We further evaluated the effect of PKC on tonic GABA_A_R activity by using a cell-permeable PKC inhibitor, BIS-I. This is widely recognized and used as a highly effective inhibitor of conventional and novel PKC isoenzymes, but it can also target other kinases (Wu-Zhang & Newton, 2013). Bath application of 500 nm BIS-I gradually increased RMS noise at both room and physiological temperature (Fig. 5A–D). At room temperature, this effect was apparent within a few minutes of drug application, in contrast to a slower onset over a period of 5–10 min at physiological temperature. The increase in RMS noise became significant after 15–20 min (Table 3: room temperature, control, 3.22 ± 0.31 pA, at 15 min after BIS-I application, 3.49 ± 0.35 pA, *n* = 7, *P* = 0.015; physiological temperature, control, 3.28 ± 0.23 pA, at 15 min after BIS-I application, 3.83 ± 0.40 pA, *n* = 7, *P* = 0.01).

Application of BIC at the end of the recording blocked the BIS-I-evoked increase in noise, and further lowered RMS noise to below control levels (Fig. 5A and C). Furthermore, pre-application of BIC blocked the effect of BIS-I (Table 3: room temperature, 0.4 ± 3.9% increase in RMS noise, *n* = 9, *P* = 0.93). As before, series resistance did not account for these changes, remaining stable throughout (room temperature, control, 15.4 ± 2.0 MΩ, after BIS-I application, 15.7 ± 2.1 MΩ; physiological temperature, control, 12.9 ± 1.0 MΩ, after BIS-I application, 13.0 ± 1.0 MΩ). We also checked whether BIS-I had any effects on phasic inhibition by constructing average sIPSC waveforms. As with the PMA data, there were no effects on sIPSC parameters at either recording temperature (Fig. 5E and F; Table 2). Taken overall, these data indicate that the noise increase evoked by BIS-I is mediated by GABA_A_Rs.

### PKC regulates tonic GABA_A_R activity in thalamic dLGN relay neurons

Thalamic relay neurons within the dLGN express a tonic GABA_A_R-mediated conductance that, similar to that of hippocampal DGGCs, is likely to be mediated by α4βδ GABA_A_Rs (Cope *et al*., 2005; Bright *et al*., 2007). Given our results with tonic GABA currents in DGGCs, we next examined whether tonic inhibition in dLGN relay neurons can be modulated by a similar PKC-dependent mechanism.

**Fig. 6:**
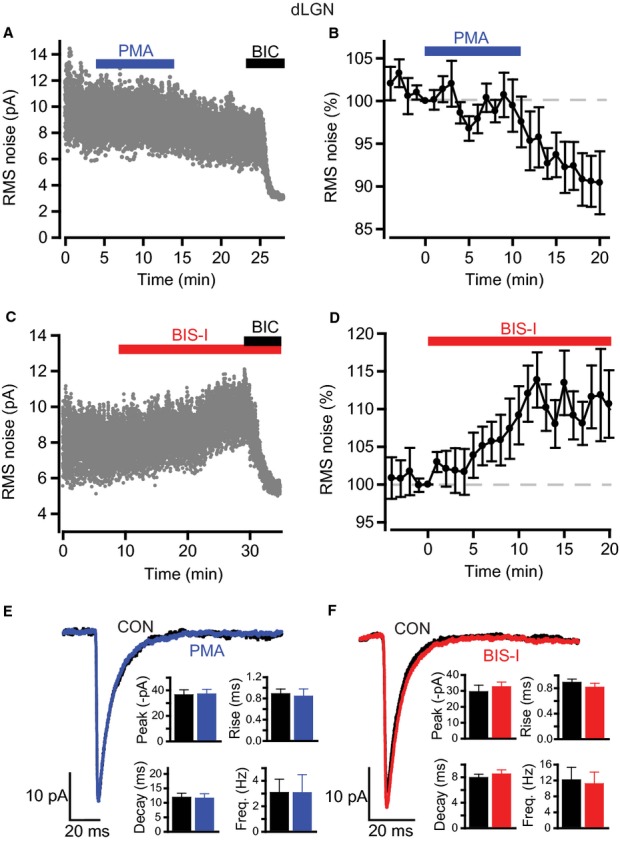
Modulation of GABA_A_ receptor-mediated inhibition by PKC in thalamic dLGN relay neurons. (A and C) RMS noise recorded from visually-identified thalamic relay neurons within the dLGN at room temperature in the presence of 200 nm PMA (A) and 500 nm BIS-I (C). PMA reduced the noise whereas inhibition of PKC with BIS-I had the reverse effect. Bicuculline reveals the extent of tonic GABA_A_ receptor activity. (B and D) Time courses for normalized data pooled from all recordings for PMA (B, *n* = 8) and BIS-I (D, *n* = 8). **(**E and F) Average sIPSC waveforms for control, and after either PMA or BIS-I. Insets: bar charts showing the lack of effect on sIPSC parameters (peak amplitude, 10–90% rise time, decay time and frequency).

Recording from visually identified relay neurons within the dLGN at room temperature showed that PMA (200 nm for 10 min) gradually reduced RMS noise (Fig. 6A and B, Table 3: control, 5.76 ± 0.74 pA; at 20 min after PMA application, 5.13 ± 0.60 pA; *n* = 8, *P* = 0.04). Application of BIC at the end of the recording revealed that there was still a component of GABA_A_R-mediated noise remaining (Fig. 6A). In contrast, inhibition of PKC with BIS-I caused a slow increase in RMS noise (Fig. 6C and D, Table 3: control, 5.04 ± 0.59 pA; at 15 min after BIS-I application, 5.73 ± 0.73 pA; *n* = 8, *P* = 0.02). As with the DGGC recordings, BIC subsequently reduced RMS noise to below control levels, indicating that the BIS-I effect is mediated purely by tonic GABA_A_R activity (Fig. 6C). Indeed, pre-application of BIC abolished the changes in noise caused by PMA (0.2 ± 4.4% decrease in RMS noise, *n* = 6, *P* = 0.75) and BIS-I (3.6 ± 1.9% decrease in RMS noise, *n* = 5, *P* = 0.14). Examination of sIPSCs showed that neither PMA nor BIS-I had any impact on phasic inhibition within these cells (Fig. 6E and F). As for the DGGCs, control recordings indicated that baseline RMS noise remained stable over a period of 25 min (normalized noise, 103.2 ± 8.4%, *n* = 5, *P* = 0.80). Thus, endogenous PKC activity within dLGN relay neurons is capable of regulating tonic inhibition in a bidirectional manner, with increased and decreased PKC function leading to diminished and enhanced tonic GABA_A_R activity, respectively.

### Modulation of recombinant δ subunit-ontaining GABA_A_Rs by protein kinases

Given that tonic GABA_A_R-mediated inhibition in hippocampal DGGCs and in thalamic dLGN relay neurons can be modulated in a similar manner by PKC, it is of interest that tonic inhibition in these two cell types is likely to be mediated predominantly by the same extrasynaptic GABA_A_R subtype, i.e., α4β2δ (Pirker *et al*., 2000; Cope *et al*., 2005; Glykys & Mody, 2007; Herd *et al*., 2008).

**Fig. 7:**
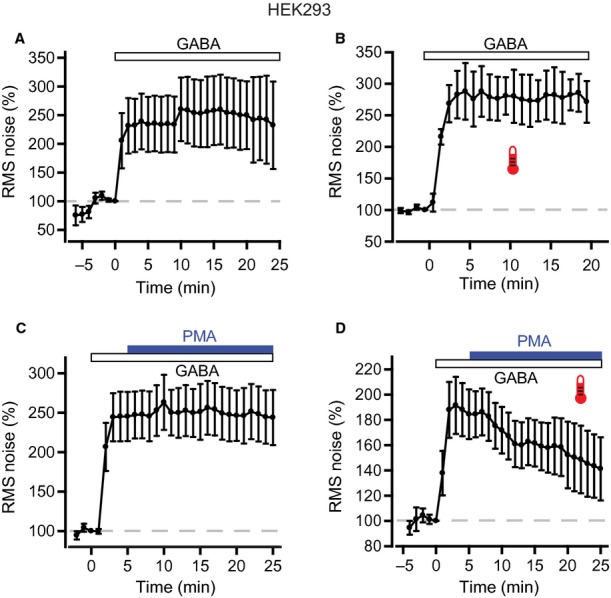
Temperature-dependent modulation of recombinant α4β2δ GABA_A_Rs by PKC. (A) Whole-cell recordings from HEK293 cells expressing α4β2δ GABA_A_Rs. Bath application of a low concentration of GABA (100 nm) was used to reproduce the physiological mode of activation of native GABA_A_Rs. RMS noise was averaged over 60-s epochs, and then normalized to the epoch just prior to GABA application. Pooled data from all cells recorded at room temperature (*n* = 6) show a sustained increase in RMS noise produced by 100 nm GABA. (B) At physiological temperature, 300 nm GABA was used to activate α4β2δ GABA_A_Rs. Pooled data (*n* = 6), normalized as above, show that this concentration of GABA led to a similar level of sustained activation as 100 nm GABA at room temperature. (C) PMA was applied 5 min after GABA application. Pooled normalized data (*n* = 9) show that PMA (200 nm) had no effect on the RMS noise evoked by GABA activation of α4β2δ GABA_A_Rs at room temperature. (D) In contrast to the room temperature experiments, at physiological temperature, application of PMA triggered a rapid decrease in GABA-activated noise (pooled data, *n* = 6).

To corroborate our findings from brain slice recordings, we investigated the modulation by protein kinases of recombinant α4β2δ GABA_A_Rs heterologously expressed in HEK293 cells. GABA was bath applied to reproduce the physiological mode of activation of these extrasynaptic receptors by relatively slowly changing, low concentrations of neurotransmitter, and recordings were made at room temperature (21–23 °C) and near-physiological temperature (34.5 ± 0.1 °C, *n* = 85). GABA concentrations were chosen to provide prolonged, sustained increases in channel activity, similar to tonic currents in neurons, enabling the co-application of kinase-modulating drugs. At room temperature, 100 nm GABA caused a significant increase in RMS noise that quickly attained a steady state and remained stable for 20–30 min (Fig. 7A: normalized response, 233.9 ± 48.5% at 5 min after GABA application, 232.4 ± 98.4% at 25 min after GABA application, *n* = 6). At physiological temperature, a slightly higher concentration of GABA (300 nm) was needed to achieve a similar level of sustained activation (Fig. 7B: normalized response, 282.7 ± 37.7% at 5 min after GABA application, 271.3 ± 33.0% at 20 min after GABA application, *n* = 6).

**Fig. 8:**
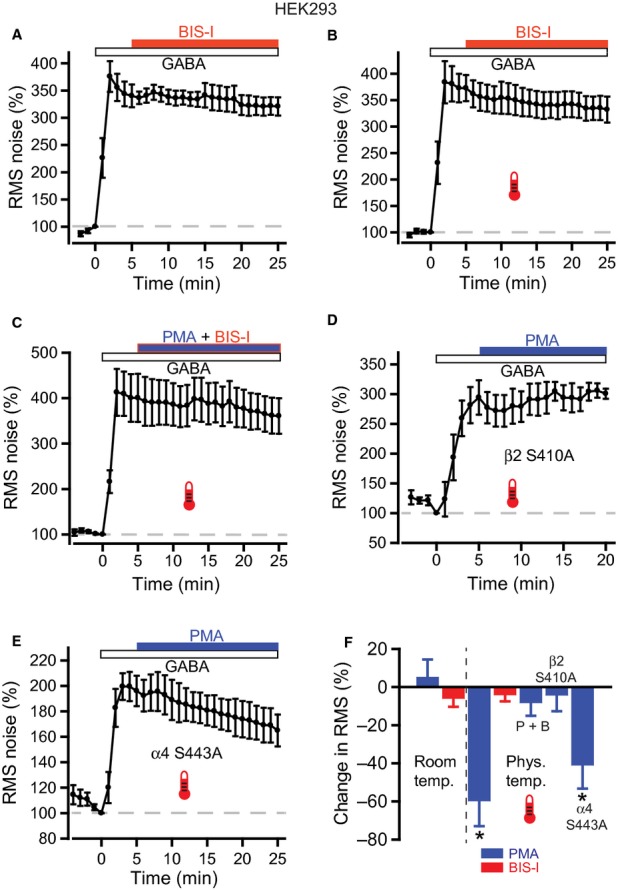
PKC inhibition of α4β2δ GABA_A_ receptor activity is mediated by phosphorylation of the β subunit. (A) RMS noise-time plot for BIS-I applied to recombinant α4β2δ GABA_A_ receptors expressed in HEK293 cells. Pooled data for room temperature experiments (n = 6) show no effect of BIS-I (500 nm) on the noise evoked by 100 nm GABA. (B) Similarly, at physiological temperature there was no significant effect of BIS-I on normalized RMS noise levels in the presence of 300 nm GABA (n = 6). (C) Effect of co-applying PMA (200 nm) with BIS-I (500 nm) at physiological temperature on RMS noise (*n* = 7) indicating that the PMA-evoked reduction in noise is due to up-regulation of endogenous PKC activity. (D) Time course for RMS noise during PMA application to mutated α4β2^S410A^δ receptors (n = 7). (E) Time course for RMS noise during PMA application to mutated α4^S443A^β2δ receptors (*n* = 7). (F) Summary plot of PMA, BIS-I and PMA + BIS-I (P + B) effects on normalized RMS noise for wild-type α4β2δ and mutant (α4β2^S410A^δ, α4^S443A^β2δ) receptors. * indicates *P* < 0.05 compared with GABA alone.

PMA (200 nm) applied 5 min after GABA did not affect the GABA-activated noise increase at room temperature (Fig. 7C: normalized response, 245.3 ± 31.1% at 5 min after PMA application, 243.9 ± 34.9% at 20 min after PMA application, *n* = 9, *P* = 0.13). In contrast, at physiological temperature, PMA progressively reduced the noise evoked by 300 nm GABA (Fig. 7D: normalized response, 184.5 ± 19.6% at 5 min after PMA application, 141.2 ± 25.0% at 20 min after PMA application, *n* = 6, equivalent to a 59.8 ± 13.2% reduction; *P* = 0.01; Fig. 8F). Hence, activation of PKC by PMA reduced the activity of α4β2δ GABA_A_Rs, but only at physiological temperature.

The effect of inhibiting PKC on δ subunit-containing GABA_A_R activity was examined at room and physiological temperatures with BIS-I (500 nm). However, no effects were observed on GABA-evoked noise at either temperature (Fig. 8A and B: room temperature, normalized response, 340.3 ± 20.9% at 5 min after BIS-I application, 320.9 ± 16.7% at 20 min after BIS-I application, *n* = 6, *P* = 0.16; physiological temperature, normalized response, 380.3 ± 25.4% at 5 min after BIS-I application, 338.5 ± 25.2% at 20 min after BIS-I application, *n* = 6, *P* = 0.13).

To establish that the large reduction in noise seen with PMA was caused by a direct effect on PKC and not by a non-specific effect on the α4β2δ GABA_A_R, we co-applied PMA with BIS-I at physiological temperature (Fig. 8C). Under these conditions, the PMA effect was blocked (normalized response, 400.6 ± 53.1% at 5 min after PMA and BIS-I co-application, 360.7 ± 39.1% at 20 min after drug application, *n* = 7, *P* = 0.21), suggesting that the PMA effect is mediated by activation of endogenous PKC in HEK cells.

The function and trafficking of GABA_A_Rs can be modulated by phosphorylation of key residues, mainly within the intracellular loops of β1–3 and γ2 subunits (Moss & Smart, 1996; Kittler & Moss, 2003). A conserved serine in receptor β subunits (Ser409 in β1 and β3; Ser410 in β2) is particularly important for modulation by PKC (Kittler & Moss, 2003). To assess whether Ser410 is involved in the PMA effect seen here, we expressed αβδ GABA_A_Rs containing a mutated β2 subunit where Ser410 is replaced with a non-phosphorylated alanine, β2^S410A^. Importantly, activation of α4β2^S410A^δ GABA_A_Rs by 300 nm GABA at physiological temperature evoked similar changes in holding current and RMS noise to those seen with wild-type α4β2δ GABA_A_Rs (GABA-activated current, α4β2^S410A^δ, –137.5 ± 23.9 pA, *n* = 7, α4β2δ, –124.2 ± 20.4 pA, *n* = 6, *P* = 0.68; GABA-activated RMS noise, α4β2^S410A^δ, 9.50 ± 1.15 pA, *n* = 7, α4β2δ, 8.30 ± 0.67 pA, *n* = 6, *P* = 0.39; unpaired *t*-tests with Welch correction), suggesting that functional receptor expression is not altered by the serine mutation. Applying PMA to α4β2^S410A^δ GABA_A_Rs failed to affect the current noise evoked by 300 nm GABA (Fig. 8D: normalized response, 294.4 ± 29.0% at 5 min after PMA application, 300.7 ± 8.5% at 15 min after PMA application, *n* = 7, *P* = 0.4). This strongly indicates that the PMA-dependent decrease in noise is caused by upregulation of PKC activity that leads to increased phosphorylation at Ser410 on β2 subunits.

A novel site on the α4 subunit, Ser443, has also been identified as an important substrate for PKC modulation of recombinant α4β3 GABA_A_Rs (Abramian *et al*., 2010). We tested the involvement of this residue by expressing GABA_A_Rs containing a mutated α4 subunit where Ser443 is replaced by an alanine, α4^S443A^. Introduction of this mutation had no apparent effect on receptor function and expression, as 300 nm GABA-evoked current and noise at physiological temperature was comparable to that observed with wild-type α4β2δ GABA_A_Rs (GABA-activated current, α4^S443A^β2δ, –157.4 ± 19.4 pA, *n* = 7, α4β2δ, –124.2 ± 20.4 pA, *n* = 6, *P* = 0.27; GABA-activated RMS noise, α4^S443A^β2δ, 6.56 ± 0.68 pA, *n* = 7, α4β2δ, 8.30 ± 0.67 pA, *n* = 6, *P* = 0.1; unpaired *t*-tests with Welch correction). Application of 200 nm PMA to α4^S443A^β2δ GABA_A_Rs resulted in a progressive decline in GABA-evoked noise, similar to that seen with wild-type GABA_A_Rs (Fig. 8E: normalized response, 196.1 ± 11.9% at 5 min after PMA application, 165.0 ± 12.5% at 20 min after PMA application, *n* = 7, *P* = 0.01). Normalization to the GABA-evoked response showed that PMA caused a 41 ± 12.3% reduction, which was similar to that seen with wild-type GABA_A_Rs (Mann–Whitney, *P* = 0.25; Fig. 8F). This indicates that, for the α4β2δ GABA_A_R used here, PKC-mediated phosphorylation of α4^S443A^ is unlikely to play a role in the PMA-dependent decrease in GABA-evoked noise.

### Activation of PKC reduces surface expression of recombinant δ subunit-containing GABA_A_Rs

**Fig. 9:**
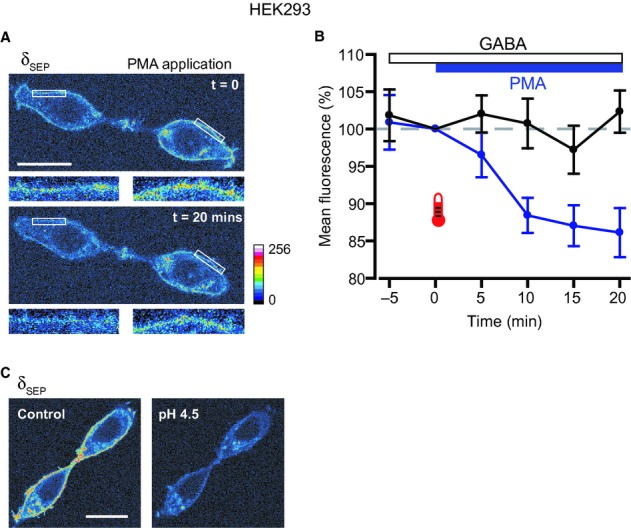
PKC activation reduces surface expression of α4β2δ GABA_A_Rs. (A) Confocal images of live HEK293 cells expressing α4β2δ-SEP GABA_A_Rs, showing the effect of PMA on surface receptor expression. GABA (300 nm) was bath applied for 5 min, before co-application of PMA (200 nm). Images obtained at the time of application (*t* = 0) and after 20 min of exposure to PMA are shown. Intensity is represented by a 16-colour spectrum (lower right). The insets below the main panels show two cell surface ROIs (white boxes) at higher magnification, and illustrate the reduction in fluorescence intensity caused by PMA. (B) Pooled normalized data (*n* = 10) show the reduction in mean cell surface fluorescence caused by PMA (blue symbols and lines). In contrast, in experiments where GABA was applied alone (*n* = 10), cell surface fluorescence remained stable (black symbols and lines). The times of application for GABA and PMA are indicated by the bars. (C) Images of HEK293 cells showing quenching of surface δ-SEP fluorescence by application of a pH 4.5 solution. Note that intracellular δ-SEP fluorescence was preserved. Colour spectrum as in (A). Scale bars: 20 μm.

The PMA-evoked reduction in δ subunit GABA_A_R activity could be caused by inhibition of channel function and/or a decrease in surface receptor expression. To address this issue, we used live imaging of HEK293 cells expressing α4β2δ–SEP GABA_A_Rs. The SEP moiety is a variant of green fluorescent protein that shows pH-sensitive fluorescence, showing very little emission at pH < 6.0 (Ashby *et al*., 2004). Hence, SEP-tagged proteins show bright fluorescence at the cell surface, but limited fluorescence in intracellular compartments, where the pH is lower. HEK293 cells expressing α4β2δ–SEP GABA_A_Rs were imaged at 30–32 °C, during bath application of GABA (300 nm), followed by co-application of PMA (200 nm). Application of PMA caused a progressive reduction in cell surface fluorescence that appeared to plateau after 15–20 min (Fig. 9A and B: normalized fluorescence at *t* = 20 min, 86.1 ± 3.3%, *n* = 10, *P* = 0.004). In contrast, during control experiments in which GABA was applied alone, surface fluorescence remained stable (Fig. 9B: normalized fluorescence at *t* = 20 min, 102.3 ± 2.8%, *n* = 10, *P* = 0.31). Surface fluorescence could be quenched by bath application of a pH 4.5 solution (Fig. 9C). Therefore, PMA-evoked activation of PKC causes a decrease in cell surface expression of α4β2δ GABA_A_Rs that correlates well with the reduction in channel activity observed during current recordings.

## Discussion

DGGCs play a vital role in hippocampal information processing by filtering out synchronous excitatory activity and preventing the generation of seizures in downstream hippocampal structures (Heinemann *et al*., 1992). Key to this role is the low excitability of these cells, part of which is derived from the efficacy and timing of GABA-mediated inhibition in the DG (Coulter & Carlson, 2007). In comparison, thalamic relay neurons within the dLGN process visual inputs from the retina, before passing this information onto the cortex. Inhibitory input to these cells is critically involved in visual information processing, modulation of receptive field properties, signal selectivity, information encoding, and spike firing mode behaviour (Hubel & Wiesel, 1961; Sillito & Kemp, 1983; Holdefer *et al*., 1989; Wang *et al*., 2007, 2011). Both dentate and thalamic cell types express populations of synaptic and extrasynaptic GABA_A_Rs to provide phasic and tonic inhibition (Nusser & Mody, 2002; Cope *et al*., 2005; Coulter & Carlson, 2007). The tonic component is considered to be a key regulator of excitability in both neuronal populations (Nusser & Mody, 2002; Cope *et al*., 2005; Maguire *et al*., 2005; Bright *et al*., 2007; Coulter & Carlson, 2007). Thus, elucidating how tonic inhibition is temporally modulated is important for understanding information processing in the hippocampus and thalamus.

To explore whether tonic inhibition can be dynamically regulated, we used slice preparations from older animals in which the molecular components underpinning GABAergic transmission, including those controlling GABA release, postsynaptic receptor expression, and GABA uptake, have matured. Recording temperature affects both GABA release and GABA uptake, and is therefore vital for setting the extent of tonic inhibition (Glykys & Mody, 2007); hence, recordings were performed at both room temperature (21–23 °C) and physiological temperature (35–37 °C). The concentration of GABA within the slice is another key determinant of tonic GABA current, and some investigators add extra GABA or GABA uptake blockers to boost the tonic current amplitude. However, we did not do this, because: increased GABA levels may recruit additional populations of receptors (Scimemi *et al*., 2005); GABA uptake as such is vital for regulating tonic GABA currents within these cells (Nusser & Mody, 2002; Cope *et al*., 2009); and extrasynaptic GABA_A_Rs are strongly desensitized when exposed to increasing ambient GABA levels (Mortensen *et al*., 2010; Bright *et al*., 2011). Thus, although we have adopted a ‘physiological’ approach, it confers a disadvantage in that tonic current amplitudes are relatively small, particularly in the DG at room temperature (8.6 ± 2.1 pA, *n* = 22). To obviate this, we used changes to RMS baseline noise to reliably monitor tonic inhibition. This approach was validated with zolpidem and THDOC to selectively potentiate the function of synaptic γ2 and extrasynaptic δ subunit-containing GABA_A_Rs, respectively (Nusser & Mody, 2002; Stell *et al*., 2003). Consistent with their selectivity, zolpidem increased the peak amplitude and decay time of sIPSCs without affecting RMS noise, whereas THDOC only increased the RMS noise. Thus, drugs that potentiated (THDOC) and inhibited (BIC) extrasynaptic GABA_A_Rs increased and reduced RMS noise, respectively, with a sensitivity that could not be matched by measuring holding currents alone. Therefore, we can be confident that, in our system, changes in RMS noise are positively correlated with changes in extrasynaptic GABA_A_R activity.

With the use of RMS noise, the selective activation of PKC was shown to decrease tonic GABA_A_R activity in DGGCs at both room and physiological temperature. In contrast, increased tonic receptor activity was apparent after inhibition of PKC with BIS-I. In both cases, no effects were observed on phasic inhibition in the form of sIPSCs. However, consistent with a previous study using intracellular application of PKC in DGGCs, we saw an increase in the peak amplitude of mIPSCs after PMA application (Poisbeau *et al*., 1999). It is unclear why we observed an effect of PKC activation on the amplitude of mIPSCs, but not sIPSCs, although the earlier study would suggest a postsynaptic effect, probably via enhanced receptor function. The differential modulation of the two forms of inhibition (tonic and phasic) could be attributable to the expression of different β subunit isoforms in the underlying receptor populations. Herd *et al*. (2008) reported that synaptic receptors are likely to contain β3 (and possibly β1) subunits, whereas the benzodiazepine-insensitive extrasynaptic δ subunit-containing GABA_A_Rs mediating the majority of tonic inhibition probably contain the β2 subunit. As it has been shown that the identity of the β subunit is critical for phospho-dependent regulation (McDonald *et al*., 1998; Nusser *et al*., 1999; Brandon *et al*., 2003; Houston *et al*., 2007), differential β subunit expression may be responsible for the disparate regulation of tonic and phasic inhibition that we observed here.

Our investigation of tonic inhibition in dLGN relay neurons revealed similar phospho-dependent modulation, with PKC activation and inhibition causing decreases and increases, respectively, in extrasynaptic GABA_A_R activity, without affecting phasic inhibition. Although the available evidence does not unequivocally identify the GABA_A_R populations within this nucleus, it appears likely that synaptic α1β2γ2 and extrasynaptic α4β2δ GABA_A_Rs are expressed. This deduction is based on both immunohistochemical data (Sur *et al*., 1999; Pirker *et al*., 2000) and pharmacological characterization of GABA_A_R-mediated currents in the dLGN and ventrobasal thalamus (Belelli *et al*., 2005; Cope *et al*., 2005; Peden *et al*., 2008). Therefore, the β2 subunit appears to be common among thalamic GABA_A_Rs, suggesting that the differential regulation of tonic and phasic inhibition in the dLGN by PKC cannot be attributable to variant β subunits. As the extrasynaptic α4β2δ GABA_A_R appears to be common to both DGGCs and dLGN relay neurons, we used heterologous expression of recombinant receptors to investigate the underlying mechanisms of PKC regulation of tonic inhibition. These experiments demonstrated that PKC activation reduced α4β2δ GABA_A_R activity at physiological temperature, while having no effect at room temperature. Live cell imaging of α4β2δ–SEP GABA_A_Rs showed that this reduction was correlated with a decrease in receptor surface expression, indicating that PKC activation causes a change in the trafficking of extrasynaptic GABA_A_Rs. In contrast, inhibition of PKC activity had no effect on α4β2δ GABA_A_R-mediated noise at either temperature.

Although very few studies have recorded from HEK293 cells at physiological temperatures, one report highlights the importance of temperature for PKC modulation of recombinant GABA_A_Rs (Machu *et al*., 2006). In this earlier study, PMA was ineffective in modulating currents mediated by α1β2γ2 GABA_A_Rs at room temperature, but caused a decrease in function at physiological temperature (35 °C), similar to the effects shown here with α4β2δ GABA_A_Rs. This, combined with our observation that BIS-I is ineffective at both temperatures, suggests that basal PKC activity is low at both temperatures, but can be enhanced by application of a phorbol ester at physiological temperature. Although the results obtained with the expression system show a contrasting temperature dependence to that shown by the brain slice data, it is perhaps not surprising that the phosphorylation state of the receptor, which will depend upon the concerted action of various kinases and phosphatases, shows differential regulation.

Interestingly, PKC can also phosphorylate Ser443 on the α4 subunit, which enhances the surface stability of α4β3 GABA_A_Rs and prevents the run-down of GABA-activated currents in HEK293 cells (Abramian *et al*., 2010). This effect relies on phosphorylation of Ser408 and/or Ser409 in the β3 subunit (Comenencia-Ortiz *et al*., 2010), and suggests that PKC-mediated phosphorylation can enhance GABA-mediated tonic currents, in contrast to the effects that we observed. However, this apparent disparity may be accounted for by the differential regulation of α4 subunit phosphorylation in β2 and β3 subunit-containing GABA_A_R, and further raises the intriguing possibility that PKC-evoked plasticity of tonic inhibition may be bidirectional, depending on the β subunit identity in the GABA_A_R. Our recombinant receptor data indicated that phosphorylation at β2 Ser410 was necessary for the PKC-evoked decrease in δ subunit-containing GABA_A_R activity, as removing the primary site for PKC phosphorylation on the β2 subunit (α4β2^S410A^δ) ablated the PMA-evoked decrease in tonic current. In contrast, our recordings from α4^S443A^β2δ GABA_A_Rs show that the decrease in tonic activity triggered by PMA is the same as in wild-type GABA_A_R, suggesting that phosphorylation at α4 Ser443 is dispensable for the PKC regulation of α4β2δ GABA_A_R seen here.

This is the first study to examine the concurrent regulation of GABA_A_R-mediated tonic and phasic inhibition within a brain slice preparation under physiological conditions of temperature and ambient GABA levels. Our results show that the tonic inhibition mediated by α4β2δ GABA_A_Rs is modulated in a similar way by PKC-dependent phosphorylation in both the hippocampus and the thalamus, whereas phasic inhibition appears to be insensitive. Although the effects of phospho-modulatory drugs on tonic inhibition appear to be small (10–20% changes in the total RMS noise), this may partly reflect the recording conditions (no added GABA or uptake blockers). Nevertheless, modest changes in an inhibitory conductance that is continuously active are likely to have an important impact on neuronal excitability (Mitchell & Silver, 2003; Bright *et al*., 2007; Jia *et al*., 2008; Song *et al*., 2011). Our study further suggests that signalling pathways that converge on PKC activation could, in principle, lead to long-term changes in the efficacy of tonic inhibition, indicating that this important form of regulation of neuronal excitability is indeed subject to dynamic plasticity-based modulation.

## Abbreviations

BIC: bicuculline
BIS-I: bisindolylmaleimide I
DG: dentate gyrus
DGGC: dentate gyrus granule cell
dLGN: dorsal lateral geniculate nucleus
GABA_A_R: GABA_A_ receptor
HEK293: human embryonic kidney 293
IPSC: inhibitory postsynaptic current
mIPSC: miniature inhibitory postsynaptic current
PKC: protein kinase C
PMA: phorbol 12-myristate 13-acetate
RMS: root mean square
ROI: region of interest
SEP: super-ecliptic phluorin
sIPSC: spontaneous inhibitory postsynaptic current
THDOC: tetrahydro-deoxycorticosterone
